# The impact of a commercial lower extremity exoskeleton on metabolic load, perceived exertion, and physiological response to a challenging military relevant task: A randomized cross-over design pilot study

**DOI:** 10.1371/journal.pone.0314613

**Published:** 2025-01-24

**Authors:** JoEllen M. Sefton, Frances K. Neal, Philip J. Agostinelli, Nicholas C. Bordonie, Phillip E. Whitley, Nathan T. Pickle, Paulien E. Roos

**Affiliations:** 1 School of Kinesiology, Auburn University, Auburn, AL, United States of America; 2 CFD Research Corporation, Huntsville, AL, United States of America; Imperial College London, UNITED KINGDOM OF GREAT BRITAIN AND NORTHERN IRELAND

## Abstract

**Purpose:**

To assess physiological metrics during the use of a commercially available bilateral active ankle exoskeleton during a challenging military-relevant task and if use of the exoskeleton during this task influences: metabolic load, physiological measures or rate of perceived exertion.

**Methods:**

Nine healthy volunteers (5M, 4F) completed this randomized cross-over design trial, with a baseline visit and two randomized test sessions (with/without the exoskeleton). Variables included impact on time to exhaustion during walking on a treadmill at varying speeds and gradients (0–15%) at 26.7°C, 50% humidity with a loaded rucksack (30% body weight). The primary outcome measure was change in metabolic cost with/without the exoskeleton (O2 consumption, metabolic equivalents); secondary outcomes were change in heart rate and perceived exertion between conditions.

**Results:**

Participants averaged 22.4 ± 4.5 years old, 173.7 ± 7.4 cm tall, weighed 80.9 ± 13.9 kg, and VO_2_max of 43.8 ± 10.6 mL/kg/min. Total kcals did not differ between conditions (with/without exoskeleton; t = 0.98; p = 0.357). Kcals/min were significantly lower (1.06 kcals/min) with the exoskeleton (t = 3.94; p = 0.004). Average oxygen consumption (VO2) was significantly lower (2.36 mL/kg/min) with the exoskeleton (t = 2.81; p = 0.023), and peak VO2 was 3.33 mL/kg/min lower with the exoskeleton (t = 2.37; p = 0.045). Peak and Average METS were also lower with the exoskeleton by 0.98 (t = 2.61; p = 0.031) and 1.23 (t = 2.39; p = 0.044) respectively.

**Conclusions:**

Results suggest a powered ankle exoskeleton may decrease energy consumption during military relevant tasks when conducted in a laboratory environment. There may also be physiological benefits such as reduced core temperate and heart rate. Replication of this work in the field environment is warranted.

## Introduction

Exoskeletons have emerged as promising technology for enhancing human strength and mobility. Over the past three decades, substantial progress has been made in developing viable systems for augmenting human strength in military-relevant tasks [1]. A range of prototype exoskeleton devices, each tailored to specific tasks, are currently in various stages of development [1–8]. Lower extremity exoskeletons hold considerable potential for enhancing military performance, particularly in scenarios involving the transportation of heavy loads across challenging terrains [9–11]. A key advantage of powered exoskeletons is their ability to decrease energy expenditure [12–15]. This reduction in energy consumption can yield several secondary benefits, including lowered core temperature, increased endurance, extended travel distances, enhanced load-carrying capacity, and potentially decreasing risk of musculoskeletal injuries [1] resulting from both physical and mental fatigue [16].

Exoskeletons exhibit considerable diversity in their design and functionality, encompassing passive and actively assisting devices, those with flexible or rigid components, and configurations spanning from single-joint to all three lower extremity joints (hip, knee, and ankle) [1]. Among these, the rigid, powered ankle exoskeleton stands out as a particularly promising design for military applications. This specific exoskeleton design boasts a compact form factor and relatively low power requirements compared to full lower extremity models, rendering it highly appealing for military use. Despite exclusively targeting the ankle, these devices have demonstrated the ability to reduce the metabolic cost of both unloaded and loaded walking by 10% and 8%, respectively [17–19]. This is achieved through the generation of substantial positive mechanical power across the ankle joint while adding minimal distal mass and preserving ankle joint degrees of freedom [17, 20]. However, previous evaluations of this exoskeleton have been confined to controlled laboratory settings. Military applications introduce a multitude of challenges, including diverse terrains, varying speeds, load carriage, and extreme environmental conditions. Consequently, the extent to which a powered ankle exoskeleton can confer benefits in these demanding conditions remains uncertain.

The purpose of this study was to assess physiological metrics during the use of a commercially available bilateral active ankle exoskeleton during a challenging military-relevant task. The research questions were: 1) does the active ankle exoskeleton extend the time to exhaustion during walking on a treadmill at varying speeds and gradients (0–15%) at 26.7°C, 50% humidity with a loaded rucksack (30% body weight); 2) does the exoskeleton influence the metabolic load of completing the task; 3) does the exoskeleton influence other physiological measures during the task; and 4) does the exoskeleton influence rate of perceived exertion during the task. We hypothesized use of the bilateral exoskeleton would: extend the time to exhaustion and reduce ratings of perceived exertion, but not influence metabolic load or physiological measures in during the military task. The findings from this study will contribute to optimizing exoskeleton design and development for future possible fielding of exoskeletons with military populations.

## Materials and methods

**Fig 1:**
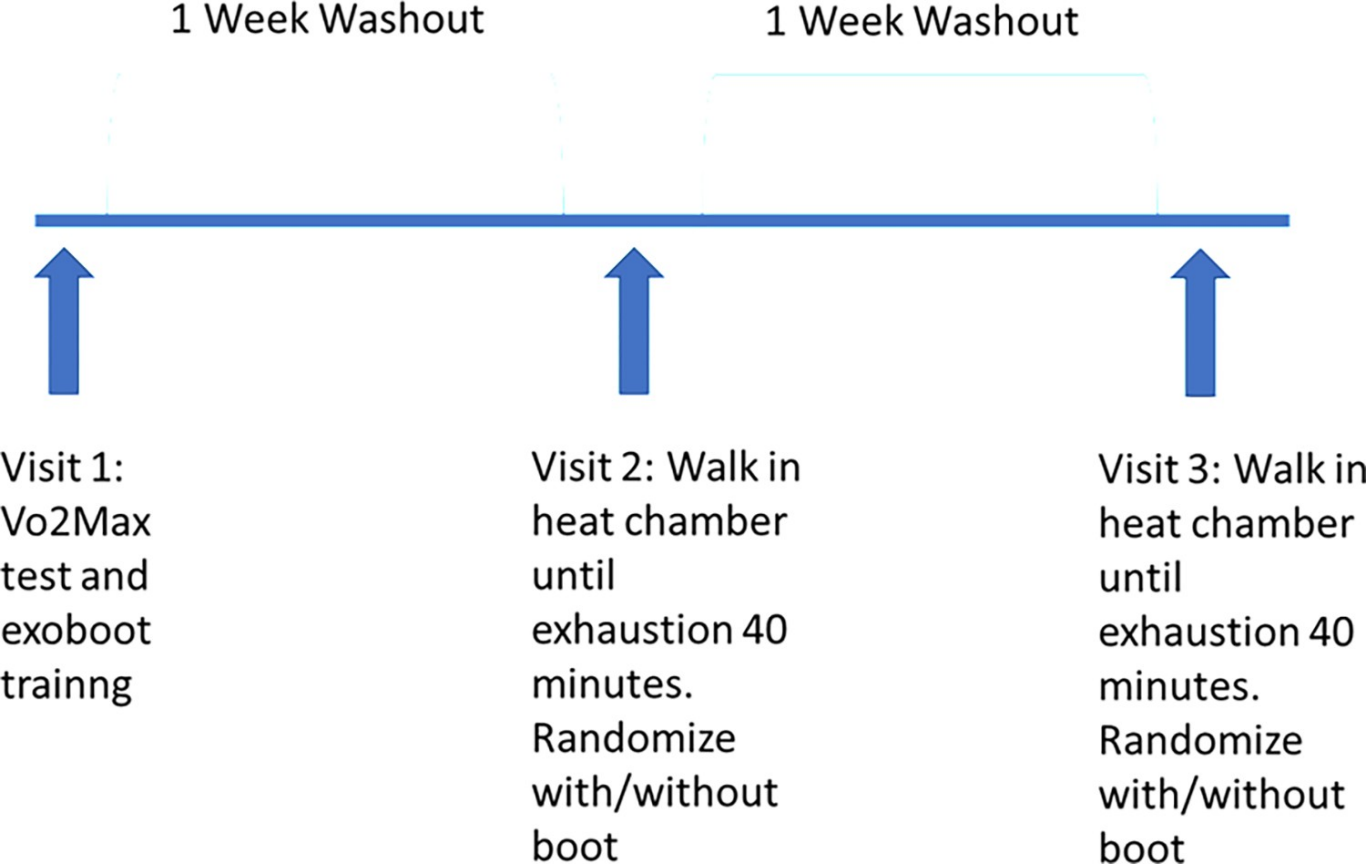
Research design.

This study was a randomized cross-over design. Participants visited the laboratory on three separate occasions each separated by a one-week wash-out period to reduce the risk of any delayed on-set muscle soreness from interfering with subsequent trials. A baseline testing session was completed during the initial visit, followed by two randomized testing sessions; one with the Exoskeleton on and one without. All sessions were separated by one week (Fig 1).

The primary outcome measure was change in metabolic cost with and without the exoskeleton. The secondary outcomes were change in physiological measures and perceived exertion between conditions. Inclusion criteria: free of musculoskeletal injury, comfortable carrying out exercise tasks at elevated temperatures, agree to adhere to study requirements, and able to pass a health screening. Exclusion criteria: known medical physical or psychological condition preventing participation in exercise; rehabilitating from recent musculoskeletal injury; diagnosed with asthma, a history of heart condition or high blood pressure; feels pain in chest at rest, during activities of daily living, or when performing physical activity or exercise; primary care physician has prescribed medically supervised physical activity only, or pregnant. Members of the research team reviewed the written informed consent with each volunteer and answered all questions. After reviewing the study requirements, inclusion and exclusion criteria and required visits, volunteers that wished to participate signed the written informed consent. Volunteers were assigned a participant number which was used for all data collection and analysis to protect the identity of the participant. Study protocols were approved by the Auburn University Institutional Review Board (protocol # 22–268 EP 2206).

Nine healthy physically active individuals (5 males and 4 females), ages of 19–35 took part in this pilot study that was part of a larger funded project to develop software to model future exoskeleton use and development. Nine individuals were screened for inclusion/exclusion criteria and boot fit, there were no dropouts. Army boots with the adaptations needed to use the ankle exoskeleton were supplied by the funder in specific sizes. These were common sizes and it was easy to find male participants that could wear the boots. They were, however, large for our female participants making it a challenge to find females that wore the larger sizes. The limited boot size availability was a limiting factor for participation. Participants completed the two exoboot sessions in randomized order to eliminate and effect of testing order.

### Baseline testing

Height and weight were assessed using a Seca scale and stadiometer (SECA, Hamburg, Germany). Age, and sex were recorded along with the following resting variables: heart rate, respiratory rate, skin temperature, and core temperature (Equivital EQ02+ LifeMonitor with BlackGhost package, Equivital, Cambridge, United Kingdom).

Maximal oxygen consumption (VO_2_max) was assessed using a standard Bruce protocol [21] for treadmill graded exercise testing to understand baseline aerobic fitness and quantify relative intensity during the exercise protocols. The VO_2_max protocol involved incremental increases in speed and incline every three minutes after one warm-up stage. The warm-up stage began at 2.74 mph and 0% incline. For the first stage after the warm-up speed remained the same and incline was increased to 10%. Each subsequent stage involved a sequential increase in both speed and incline. Participants were asked to give their best effort and continue until they could not continue any longer. The test was terminated once participants reach volitional fatigue. VO_2_max was determined as the maximal oxygen consumption achieved during the test. Criteria to verify a valid maximal effort was achieved by the participant included: a respiratory exchange ratio (RER) >1.1, Heart rate peak within 10 bpm of age predicted max heart rate, and a plateau in VO_2_.

After pre-testing participants practiced the test protocol wearing the EB60 ExoBoot exoskeleton (Dephy, Inc. Boxboro, MA USA) bilaterally for 10–15 minutes or until they felt comfortable.

### Load carriage protocol

**Fig 2:**
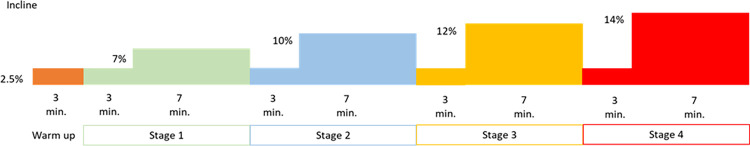
Diagram of exercise intervention progression.

**Fig 3:**
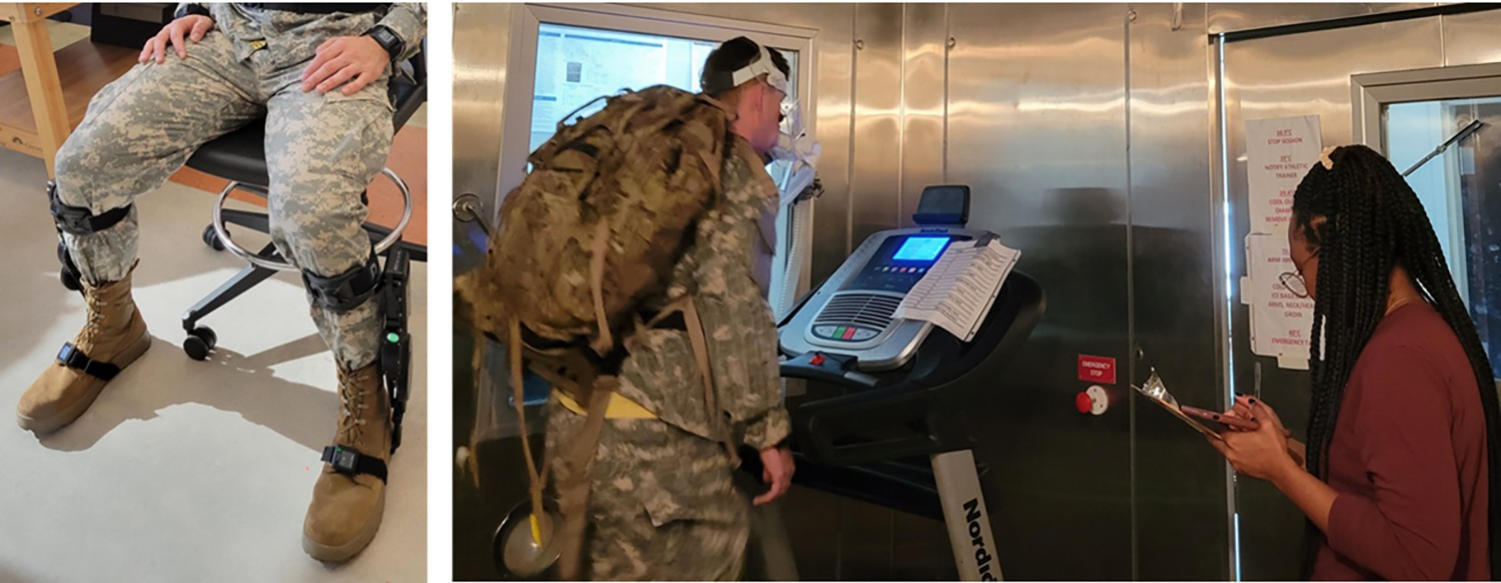
Participant performing the Load carriage protocol.

Participants were asked to walk until exhaustion or for 40 minutes on a treadmill at varying speeds and elevations in an environmental chamber set to 26.7°C (80°F) at 50% relative humidity. (ESPEC North America, Inc., Hudsonville, MI, USA). Participants walked on the treadmill at three miles per hour. The testing protocol began with a warm-up at 2.5% incline for three minutes. Stage 1 involved walking for three minutes at a 2.5% incline, followed by seven minutes at a 7% incline. Stage 2 included three minutes at a 2.5% incline, followed by seven minutes at a 10% incline. Stage 3 involved three minutes at a 2.5% incline, followed by seven minutes at a 12% incline. Finally Stage 4 included three minutes at a 2.5% incline, followed by seven minutes at a 14% incline (Fig 1). Participants completed as much of the testing protocol as possible (Figs 2 and 3). The protocol was terminated when the participant reached a core temperature of 38.9°C, reached exhaustion, or indicated they wanted to stop. This protocol was standardized for both conditions (the exoboot and without the exoboot) and conditions were separated by a seven-day wash-out period to minimize potential delayed on-set effects of previous trials.

### Measures

During both VO2max testing and experimental trials ventilation and expired gases were measured using the ParvoMedics True One 2400 metabolic cart (ParvoMedics Inc. Salt Lake City, UT, USA). Values obtained from the metabolic cart included oxygen consumption (VO_2_), kilocalories (kcals) and Metabolic Equivalents (METs). The gas analyzer was calibrated for volume (Hans Rudolph Series 5530 3L syringe; Shawnee, KS, USA) and gas composition (16% O_2_ and 4% CO_2_) prior to each participant testing session. Participants were fitted with a head gear, mouthpiece, and nose clip (Hans Rudolph, Inc.; Shawnee, KS, USA) for oxygen measurements. During performance heart rate was monitored continuously via the Equivital EQ02+ LifeMonitor and Black Ghost Software (Hidalgo, Cambridge, UK).

Rate of perceived exertion (RPE) was collected during the VO2 max testing using a printed OMNI (0–10) scale. Change in RPE was calculated as the difference between baseline and final RPE.

### Statistical analysis

The Shapiro-Wilks Test was used to evaluate normality. A dependent t-test was performed to compare conditions. The independent variable was condition (with and without the active ankle exoskeleton); the dependent variables included measures of metabolic cost (kcals/min, relative VO2, and METS), physiological strain (heart rate and core temperature), and perceived exertion (RPE) using the OMNI 0–10 rate of perceived exertion scale [22]. Most data is expressed as mean ± standard deviation. Due to the ordinal nature of the RPE data and the relatively small sample size (n = 9), non-parametric Wilcoxon signed-rank tests were conducted to compare RPE measures (delta, peak, and average) between exoskeleton and no-exoskeleton conditions. Effect sizes (r) were calculated from the Wilcoxon test statistics and interpreted as negligible (r < 0.1), small (0.1 ≤ r < 0.3), medium (0.3 ≤ r < 0.5), or large (r ≥ 0.5). Multiple participants experienced non-fatigue related termination of the protocol (pain or discomfort experienced from the boot or ruck, see discussion below), thus a second follow-up analysis was completed time-matching each participant’s trials to each other to provide a more accurate an equal comparison of metabolic cost experienced and physiological strain. Time matching was done by taking the longer trial time to complete and trimming the other sessions data to the time. This removed time to exhaustion as an outcome variable but assured that the participants completed the same amount of the protocol and therefore the same exertional stress response (Tables 5–7). This analysis was completed using the same statistical methods as the original data. Significance was set *a priori* at a *p* ≤0.05 for all measurements. R Statistical Programming Software Version 4.1.2 (RStudio; Boston, MA, USA) was used for all statistical analyses.

## Results

Nine participants were screened and completed this pilot study. All 9 participants (5 males and 4 females) completed baseline testing and both experimental trials. Participants were on average 22.4 ± 4.5 years old, 173.7 ± 7.4 cm tall, weighed 80.9 ± 13.9 kg, and had a VO_2_max of 43.8 ± 10.6 mL/kg/min. Additional descriptive statistics including minimum, maximum and range values are included in Table 1. All variables of interested met assumptions of normality.

**Table 1:** Descriptive statistics.

Variable	Mean ± SD	Min	Max	Range
Age (yrs)	22.4 ± 4.5	19.0	34.0	15.0
Height (cm)	173.7 ± 7.5	162.5	187.0	24.5
Weight (kg)	80.9 ± 13.9	63.7	104.2	40.5
VO_2_max (mL/kg/min)	43.8 ± 10.6	29.4	58.4	29.0

Notes: yrs = years; cm = centimeters; kg = kilograms

In assessing metabolic cost, total kcals did not differ significantly between conditions (with or without the active ankle exoskeleton) (t = 0.98; p = 0.357). However, Kcals/min were significantly lower (1.06 kcals/min) with the exoskeleton (t = 3.94; p = 0.004). Average oxygen consumption (VO2) was significantly lower (2.36 mL/kg/min) with the exoskeleton (t = 2.81; p = 0.023), and peak VO2 was 3.33 mL/kg/min lower with the exoskeleton (t = 2.37; p = 0.045). Peak and Average METS were also lower with the exoskeleton by 0.98 (t = 2.61; p = 0.031) and 1.23 (t = 2.39; p = 0.044) respectively. Mean and standard deviations for these variables are displayed in Table 2.

**Table 2:** Metabolic cost.

Variable	Without Exoskeleton (Mean ± SD)	With Exoskeleton (Mean ± SD)	p-values	Percent Difference ((Without–With Exoskeleton)/Without)
Total Kilocalories	272.78 ± 145.70	242. 56 ± 132.48	p = 0.357	
Kilocalories/min	10.80 ± 1.88	9.74 ± 1.84	p = 0.004*	9.8%
Avg VO2 (%max)	26.28 ± 3.32 (62%)	23.92 ± 3.10 (56%)	p = 0.023*	9.0%
Peak VO2 (%max)	36.63 ± 7.41 (85%)	33.30 ± 6.16 (78%)	p = 0.045*	9.1%
Avg METS	7.81 ± 1.30	6.83 ± 0.89	p = 0.044*	12.5%
Peak METS	10.74 ± 2.15	9.51 ± 1.75	p = 0.031*	11.5%

Notes: VO2 = Volume of Oxygen Consumed; METS = metabolic equivalents. * Indicates significant difference (p < 0.05).

Physiological strain assessments revealed peak and average heart rate were significantly lower (7.9 bpm) with the active ankle exoskeleton compared to without the exoskeleton (t = 2.4; p = 0.044). Average heart rate was significantly lower (9. bpm) with the exoskeleton (t = 2.9; p = 0.021). Average core temperature was significantly lower (0.25°C) with the exoskeleton. Participants started 0.07°C lower and ended 0.25°C lower with the exoskeleton (t = 2.20; p = 0.013). Peak core temperature was not significantly different and did not differ between the conditions (t = 2.13; p = 0.066). Mean and standard deviation values for these variables are detailed in Table 3.

**Table 3:** Physiological strain.

Variable	Without Exoskeleton (Mean ± SD)	With Exoskeleton (Mean ± SD)	p-values	Percent Difference ((Without–With Exoskeleton)/Without)
Baseline HR (BPM)	102.67 ± 15.19	96.01 ± 11.96	p = 0.096	
Peak HR (BPM)	188.67 ± 8.82	180.78 ± 16.12	p = 0.044*	4.2%
Average HR (BPM)	161.69 ± 10.76	152.44 ± 17.61	p = 0.021*	5.7%
Baseline Core Temperature (C°)	37.33 ± 0.30	37.20 ± 0.21	p = 0.035*	0.3%
Peak Core Temperature (C°)	38.56 ± 0.26	38.27 ± 0.42	p = 0.066	
Average Core Temperature (C°)	37.96 ± 0.22	37.71 ± 0.30	p = 0.013*	0.7%
Delta Core Temperature (C°)	1.22 ± 0.35	1.06 ± 0.44	p = 0.384	

Note: BPM = beats per minute; HR = heart rate; min = minutes * Indicates significant difference (p < 0.05).

Wilcoxon signed-rank tests indicated Delta RPE showed the largest mean difference (1.78 lower with the exoskeleton compared to the non-exoskeleton condition). Median decreased from 6 (no exoskeleton) to 3 (exoskeleton condition). The effect size was negligible (r = 0.98). Peak RPE decreased by an average of one point with the exoskeleton with a small effect size (r = 01.54). There was a minimal change in average RPE for both mean (0.29 difference) and median (5.5 to 5.6). There was no significant difference (p = 0.78) and negligible effect size (0.056). Results are detailed in Table 4.

**Table 4:** Perceived exertion.

Mean No Exoskeleton	SD No Exoskeleton	Mean With Exoskeleton	SD With Exoskeleton	Mean Difference	W Statistic	p-value	Effect Size (r)	Effect Size (r) Interpretation
6.111	1.764	4.333	2.398	1.778	19	0.09	0.098	Negligible
8.778	0.833	7.778	2.224	1	17	0.202	0.154	Small
5.689	0.666	5.4	1.377	0.289	20.5	0.779	0.056	Negligible
**Condition**	**Median**	**Q1**	**Q3**	**IQR**	**Min**	**Max**	**Range**	
No Exoskeleton	6	6	7	1	3	9	6	
Exoskeleton	3	2	6	4	2	8	6	
No Exoskeleton	9	9	9	0	7	10	3	
Exoskeleton	9	6	9	3	4	10	6	
No Exoskeleton	5.5	5.2	6.2	1	5	6.9	1.9	
Exoskeleton	5.6	4.3	6.3	2	3	7	4	

Follow up analysis included time-matched comparisons which indicate total caloric expenditure is not significantly different with the active ankle exoskeleton (t = 0.67; p = 0.523). Average METS were no longer significantly different when time matched (t = 1.87; p = 0.098). All other variables saw small changes that did not impact the statistical outcomes (Table 5). When time matched all physiological stain and perceived exertion variables revealed small changes that did not impact the statistical outcome (Tables 6 and 7).

**Table 5:** Time matched metabolic cost.

Variable	Without Exoskeleton (Mean ± SD)	With Exoskeleton (Mean ± SD)	p-values	Percent Difference ((Without–With Exoskeleton)/Without)
Total Kilocalories	235.44 ± 140.28	221. 67 ± 135.68	p < 0.001	
Kilocalories/min	10.58 ± 2.00	9.62 ± 1.83	p = 0.007*	9.1%
Avg VO2 (%max)	25.39 ± 3.08 (60%)	23.37 ± 3.23 (55%)	p = 0.008*	8.0%
Peak VO2 (%max)	36.63 ± 7.41 (85%)	33.30 ± 6.16 (78%)	p = 0.005*	9.1%
Avg METS	7.54 ± 1.32	6.66 ± 0.91	p = 0.075	
Peak METS	10.30 ± 2.17	9.32 ± 1.56	p = 0.049*	9.5%

Note: VO2 = Volume of Oxygen Consumed; METS = metabolic equivalents. * = statistically significant (p < 0.05).

**Table 6:** Time matched physiological strain.

Variable	Without Exoskeleton (Mean ± SD)	With Exoskeleton (Mean ± SD)	p-values	Percent Difference ((Without–With Exoskeleton)/Without)
Peak HR (bpm)	186.67 ± 12.36	179.78 ± 15.60	p = 0.008*	3.7%
Average HR (bpm)	160.09 ± 12.34	151.70 ± 17.22	p = 0.004*	5.2%
Peak Core Temperature (°C)	38.39 ± 0.29	38.09 ± 0.46	p = 0.179	
Average Core Temperature (°C)	37.97 ± 0.38	37.83 ± 0.39	p = 0.348	
Delta Core Temperature (°C)	1.06 ± 0.38	0.82 ± 0.41	p = 0.149	

Note: HR = heart rate; BPM = beats per minute; C = Celsius; * = statistically significant (p < 0.05).

**Table 7:** Individual data.

Participant	Age (yrs)	Sex (M/F)	Height (cm)	Weight (kg)	VO2Max (mL/kg/min)	Trial	Kcal/min	VO2 Avg	METs Avg	HR Avg (bpm)	CT Avg (C°)	Peak RPE	Total Time (min)	Notes
1!	20	M	173.0	70.3	57.8	No Exoskeleton	10.7	30.8	8.8	151	37.9	9	40	Experienced foot pain during the trial with the Exoskeleton. Also felt he could have completed the protocol better without the Exoskeleton, that it ‘didn’t do anything.”
						Exoskeleton	8.3	23.6	6.7	118	37.1	5	22	
2	20	M	178.0	74.3	46.6	No Exoskeleton	9.2	24.8	7.1	158	37.8	9	25	Experienced tripping from Exoskeleton
						Exoskeleton	9.1	25.1	7.2	155	37.8	9	27	
3	21	M	187.0	58.4	58.4	No Exoskeleton	14.2	30.8	8.8	142	37.8	8	40	ROTC Cadet with outstanding fitness. Only participant to complete the full 40-minute trial with and without the Exoskeleton.
						Exoskeleton	13.5	29.2	8.3	134	37.6	8	40	
4*	23	F	174.0	96.5	29.4	No Exoskeleton	12.2	22.3	6.4	169	38.0	7	9	Bad cramp during Exoskeleton session
						Exoskeleton	10.5	21.8	6.2	166	37.7	6	11	
5!	20	F	174.0	85.0	34.2	No Exoskeleton	11.6	26.7	10.2	163	37.7	9	17	Felt uncomfortable in mask–hard to breath started to panic somewhat during trial without the Exoskeleton. Exoskeleton kept cutting off unexpectedly and participant had to redo the Exoskeleton trial because the Exoskeleton data didn’t collect. Metabolic cart issues on Exoskeleton trial #2
						Exoskeleton	9.7	22.7	6.5	157	37.7	6	24	
6	19	M	171.0	63.7	52.3	No Exoskeleton	9.4	29.0	8.3	165	38.3	9	34	
						Exoskeleton	9.3	28.6	8.2	159	38.1	9	37	
7*	23	M	179.5	104.2	39.3	No Exoskeleton	12.3	23.3	6.7	179	38.3	9	18	
						Exoskeleton	11.6	22.2	6.3	177	38.1	10	17	
8*	34	F	162.5	73.7	34.8	No Exoskeleton	8.5	22.7	6.5	168	38.0	10	25	
						Exoskeleton	7.8	21.1	6.0	160	37.8	9	26	
9!	22	F	164.5	69.3	41/5	No Exoskeleton	9.2	26.1	7.5	160	37.8	9	21	Exoskeleton was too big. Participant did not eat a lot on the non-Exoskeleton trial. Participant was tripping frequently due to the Exoskeleton. Stated that she could have completed both trials much easier in her own Army boots. She didn’t like the boot design.
						Exoskeleton	8.0	21.0	6.0	146	37.5	10	12	

Due to the small pilot sample and significant variability between participants, the individual responses to each condition of our main variables of interest have been included in Table 7 to provide additional insight into the effect of the active ankle exoskeleton.

## Discussion

The purpose of this pilot study was to assess potential differences in time to exhaustion, metabolic cost, physiological responses, and perceived exertion when utilizing an active ankle exoskeleton compared to without the active ankle exoskeleton during a challenging military-relevant load carriage task. The findings from this study will contribute to determining value and optimizing exoskeleton design and development for future possible fielding of exoskeletons with military populations.

The active ankle exoskeleton decreased total energy expenditures compared to trials without the exoskeleton. This difference in metabolic cost is evidenced by approximately 9% reduction in kilocalories per minute (1.06 kcal/min) and oxygen consumption (2.36 mL/kg/min) when wearing the active ankle exoskeleton. Previous research in hopping (4–12% lower) [23], lifting, and walking (17% lower) support this finding [24, 25]. While small, this could be impactful across longer efforts. For example, if the decrease was maintained across a three-hour ruck march approximately 382 kcal could be saved. This difference would likely increase when carrying a heavier weight more closely resembling the standard fighting load. There was also a decrease in the percentage of relative max capacity. Participants were working at 55% of VO_2_max with the exoskeleton compared to 60% during the non-exoskeleton trials, which may result in a longer time to fatigue during an extended performance. Previous research found a significant increase in mean oxygen consumption when wearing a lower body exoskeleton [26]. The differences in findings may result from the increased weight or biomechanical changes resulting from the lower body exoskeleton compared to the lower extremity exoskeleton used in the current study. Interestingly participants did reach volitional fatigue, even though for this duration and intensity of exercise we would not expect typical physiological mechanisms of fatigue (i.e. a depletion of substrates or lactate/H+ ion accumulation] to occur. This may indicate limitations in the boot fit, mobility or other factors. However, this was not a specific variable assessed in this study. Other measures related to metabolic cost including peak VO_2,_ and peak METS reached during the load carriage protocol also proved to be significantly lower in the active ankle exoskeleton condition. A prior study using a passive-elastic knee-ankle exoskeleton also found a similar level (11%) of reduced metabolic cost while walking [27] and a decrease of 19% in older adults while walking with a tethered exoskeleton [28].

There was a trend toward reduced RPE with the exoskeleton, particularly for Delta RPE. However, the differences did not reach statistical significance (p = 0.090). The exoskeleton condition generally showed more response variability across all measures, suggesting individual differences in how participants responded to the intervention. Time to exhaustion did not differ, which disagreed with our hypothesis. There are an array of factors that may influence participants’ ability to continue exercising. Some of the individual factors could be causing participants to terminate exercise unrelated to exhaustion. These factors are highlighted in the individual participant table (Table 7) that provides an in-depth look at participant specific notes and comments during the pilot study, such as boot fit, style, or load carriage experience. In addition to these factors there were many day-to-day variables such as participant fueling, hydration, or sleep habits that were not controlled for in this pilot study that could influence time to exhaustion between conditions. Therefore, we completed a follow-up analysis using time-matched data and re-evaluated our findings. A combination of the full data and the time matched data likely provides the most representative interpretation of our results. Metabolic cost and physiological strain variables from the time matched data assure that participants completed the same amount of exercise, at the same intensities, for the same duration. This allows for an appropriate comparison of metabolic cost and physiological stress experienced as the exercise stress imposed on the individuals is identical, but the non-time matched evaluation allowed us to see if there were potential performance benefits of the exoskeleton. When time matched, we see very similar results and statistical differences.

Physiological strain variables were significantly lower with the active ankle exoskeleton compared to without the exoskeleton. Peak and average heart rate during the protocol were lower when wearing the active ankle exoskeleton compared to without the active ankle exoskeleton. Peak core temperature was significantly different, but average core temperature does not differ across conditions. Additionally, baseline core temperature was significantly different across conditions. The differences experienced during the protocol (average and peak core temperature) were likely influenced by a difference in baseline temperature rather than the effect of the active ankle exoskeleton. Additionally, we did not control for menstrual phase which can significantly impact resting and exercising core temperatures.

Peak exertion, average exertion, and change in perceived exertion did not differ across conditions. This suggests that while there was a reduced metabolic cost and physiological strain during the active ankle exoskeleton trials, it did not translate to a reduction in the perception of exertion experienced by participants and may help to explain the lack of significant change in time to exhaustion. This lack of change may be related to the factors mentioned above such as boot fit, lack of experience with load carriage, or differences in sleep or fueling. Thus, while there may have been a reduced metabolic and physiologic cost to the activity, it did not change perception of effort or actual trial performance in this pilot study. These results do support larger studies that control for sleep, load carriage experience and fueling are warranted.

Limitations for this pilot study include sample size; statistical power was limited and a future study in a larger population could help to elucidate if these decreases are meaningful and could result in performance changes. Additionally, exercise over a longer duration or higher intensity could aid future determinations on the clinical applications of an active ankle exoskeleton in military settings. The benefits of the active ankle exoskeleton are also highly dependent on comfort and fit. This pilot study recruited individuals that fit the available boots, rather than fitting boots to the individual. This was especially challenging in recruiting females as we were provided with only large sizes. It is also likely that a learning curve with both breathing during the metabolic testing mask and active ankle exoskeleton use exists [25]. The potential for this impacting the results should be reduced by our randomization of trial order. The impact of these factors as well as possible unexplored biomechanical changes on the performance suggests these results should be interpreted cautiously. The significant differences observed, however, do warrant future investigation into the impact the active ankle exoskeleton has on metabolic cost, physiological strain, and perceived exertion in a much larger sample size using a longer or more challenging task.

## Conclusion

Overall, the findings of this study demonstrate a powered ankle exoskeleton may decrease energy consumption during challenging military tasks. There may also be associated physiological benefits such as reduced core temperate and heart rate. However, as in previous exoskeleton studies there was high variability among individuals [26, 29]. Extended training periods may increase user proficiency and reduce variability in biomechanical and physiological outcomes [30]. Moreover, other person-specific neural, physiological, or biomechanical factors may play a role. These findings can help inform real-time use of active ankle exoskeletons by warfighters in the field through improved understanding of the impact of mechanical assistance on physiological metrics.
